# Risk Factors Associated with the Development of Atopic Sensitization in Indonesia

**DOI:** 10.1371/journal.pone.0067064

**Published:** 2013-06-19

**Authors:** Firdaus Hamid, Aprilianto E. Wiria, Linda J. Wammes, Maria M. M. Kaisar, Yenny Djuardi, Serge A. Versteeg, Sitti Wahyuni, Ronald van Ree, Erliyani Sartono, Taniawati Supali, Maria Yazdanbakhsh

**Affiliations:** 1 Department of Microbiology, Faculty of Medicine, Hasanuddin University, Makassar, Indonesia; 2 Department of Parasitology, Leiden University Medical Center, Leiden, The Netherlands; 3 Department of Parasitology, Faculty of Medicine, University of Indonesia, Jakarta, Indonesia; 4 Department of Experimental Immunology, Academic Medical Center, Amsterdam, The Netherlands; 5 Department of Parasitology, Faculty of Medicine, Hasanuddin University, Makassar, Indonesia; 6 Department of Otorhinolaryngology, Academic Medical Center, Amsterdam, The Netherlands; Ludwig-Maximilians-University Munich, Germany

## Abstract

**Background:**

The prevalence of allergic diseases has increased not only in high income but also in low-to-middle income countries. However, risk factors for their development are still not well established, particularly in the latter.

**Objective:**

To assess prevalence and identify risk factors for sensitization to two major inhalant allergens among children from semi-urban and rural areas in Indonesia.

**Method:**

A cross-sectional survey was performed among 1,674 school children aged 5–15 years old. Information on potential risk factors and reported allergic symptoms were obtained by questionnaire. Helminth infections were assessed. Skin prick tests (SPT) were performed, total IgE as well as allergen-specific IgE for house dust mite (HDM) and cockroach were measured.

**Result:**

The prevalence of allergic skin sensitization to both aeroallergens was significantly higher in the semi-urban than in the rural area. However, serum IgE against HDM and cockroach as well as total IgE were significantly lower in semi-urban than in rural children. In the semi-urban area, there was a significant positive association between SPT to HDM and higher paternal education but a negative one with hookworm infection. The risk factors linked to cockroach sensitization were different: being of a farmer offspring and lacking access to piped water were associated with an increased risk for a positive SPT to cockroach. No significant associations between measured risk factors and having a positive SPT were found in the rural area.

**Conclusion:**

Sensitization to HDM and cockroach is common in Indonesia, more often translating into a positive SPT in the semi-urban than in the rural setting. Whereas high paternal education and low hookworm infection were associated with increased risk of SPT to HDM, we were surprised to find parameters of lower SES were identified as risk factor for cockroach SPT.

## Introduction

The prevalence of allergic diseases has increased not only in high income but also in low-to-middle income countries (LMIC) [1]. However, in the LMIC, whereas high prevalence of allergies is seen in urban centers, in rural areas allergic disorders are usually rare [2], [3].

It is well-established that the development of allergic disorders is the result of a complex interplay of genetic background and environmental factors [4]. Various exogenous factors including parental smoking, allergen exposure, and outdoor and indoor air pollution have been shown to be associated with an increase in the prevalence of allergic disease, while other factors such as exposure to infections in early childhood, a ‘traditional’ diet or lifestyle, and contact with animals were shown to reduce the risk of developing allergic disorders [5].

With respect to infections, parasitic helminths have often been shown to be negatively associated with allergic outcomes [6]. Previous studies have shown that having *Schistosoma* [7] or filarial infection [8] decrease the risk of skin prick test (SPT) positivity. Similarly, studies in Ecuadorian [9] and Vietnamese [10] children showed that having soil-transmitted helminth infections decrease the risk of atopy. In these studies, it was also clear that other factors such as overcrowding in household, poor sanitation as well as low socioeconomic status can be associated with less atopy [9], [10]. In general, helminth infections are associated with poor sanitation, low socioeconomic status and rural setting in low income countries [11], therefore dissecting the relative contribution of the different factors will be important in understanding the rise in allergies worldwide [12].

Indonesia is one of the LMIC which is in epidemiological transition with increasing level of urbanization accompanied by changing disease patterns. Risk factors for the development of allergic disorders are still not well established in Indonesia. Therefore, we set up a study in a semi-urban and a rural area on Flores Island, in Indonesia, to estimate the prevalence of allergic sensitization to two major respiratory allergen sources (house dust mite and cockroach) and to identify associated risk factors.

## Methods

### Study area and design

This cross-sectional study was performed between January and June 2008 in Ende District of Flores Island, Indonesia, as part of a longitudinal study investigating the effect of anthelminthic treatment on malarial parasitemia and allergy, described in detail elsewhere [13], [14]. In a preliminary study, we surveyed several areas in Ende district and found that both semi-urban, Nangapanda, and rural, Anaranda, were endemic for soil-transmitted helminth. A total of 1674 (1161 and 513 from semi-urban and rural, respectively) school children aged 5 – 15 years were included in this study. Allergic disorders were assessed by a questionnaire slightly modified from the validated questionnaire used by the International Study of Asthma and Allergy in Childhood (ISAAC) to accommodate local study requirements. Questionnaires were administered to obtain demographic data, socioeconomic status and other potential risk or protective factors for the development of allergies. In addition, skin reactivity (by SPT) and specific IgE (by ImmunoCAP) to house dust mite (HDM) and cockroach as well as total IgE (by ELISA) were measured. Stool was collected for determination of helminth infection. Written informed consent was obtained from parent or guardian of each child. The study was approved by the Ethical Committee of the Medical Faculty, University of Indonesia, Jakarta (ethical clearance ref: 194/PT02.FK/Etik/2006) and has been filed by ethics committee of the Leiden University Medical Center, The Netherlands.

### Questionnaires

Reported clinical symptoms of allergy were obtained by questionnaire. The interview was conducted with the parent or guardian of the children. History of asthma and atopic dermatitis (eczema) were assessed using a modified ISAAC questionnaire, which had been translated into Bahasa Indonesia. The prevalence of asthma symptoms were obtained from the following questions: (i) Has your child ever had wheeze? (ii) Has this asthma been diagnosed by a doctor? and (iii) Has your child ever or in the past 12 months had wheezing or whistling in the chest?; while eczema was obtained from the questions: (i) has your child ever had doctor diagnosed allergic eczema and (ii) has your child ever had one or more skin problems accompanied by an itchy rash?

An additional questionnaire was administered to obtain data on demographic, parental education, parental occupation, water supply, living condition of families and other characteristics of the households. Educational levels were categorized as: low for illiterate or elementary school and high for secondary school or above. The majority reported parental occupation as ‘farmers’; therefore occupation was categorized into farmers and non-farmers.

### Skin prick testing

SPT reactivity to common aeroallergens was tested with extracts of HDM (*Dermatophagoides pteronyssinus* and *D. farinae;* HAL Allergy Laboratories, Leiden, The Netherlands) and cockroach (*Blatella germanica*; Lofarma, Milan, Italy). Histamine and allergen diluents were used as positive and negative controls, respectively. SPT was done on the volar side of the lower arm. The wheal size was measured after 15 minutes. Skin prick reactivity was considered to be positive if the longest diameter of the wheal size plus the diameter perpendicular to it divided by two was at least 3 mm. Individuals with at least one positive reaction to either *D. pteronyssinus* or *D. farinae* were classified to be positive for SPT to HDM. The same investigator performed all the SPTs. Body weight and height were also measured.

### Specific and Total IgE

The levels of specific IgE (sIgE) to *D. pteronyssinus* and *B. germanica* were measured in plasma using an ImmunoCAP 250 system, (Thermo Fisher Scientfic, Uppsala, Sweden) following the manufacturer’s instructions. All measurements were conducted in one laboratory in The Netherlands.

The levels of total IgE were measured by ELISA in Jakarta as described previously [13], [14]. The results were expressed in International Units (IU/ml).

### Parasitological examination

The formol-ether acetate concentration method was performed on formalin-preserved stool samples followed by microscopic examination for detection of *Trichuris trichiura*. Aliquots of unpreserved stool samples were kept frozen at −20°C. DNA detection of the parasite in the stool samples was performed in the Netherlands. A multiplex real-time PCR was used for the specific DNA amplification and detection of hookworm (*Ancylostoma duodenale* and *Necator americanus), Ascaris lumbricoides and Strongyloides stercoralis* as have been describes in detail previously [13]. PCR output was expressed as the cycle threshold (Ct) reflecting the load of parasite specific DNA in the sample tested. Parasite specific DNA loads of *N. americanus* and *A. lumbricoides* were categorized in low load (Ct≥30) and high load (Ct<30).

### Statistical analysis

We investigated risk factors for allergic disorders separately for each area. Descriptive data were expressed as means (± standard deviations), geometric means [95% confidence intervals (CI)] and frequencies (percentage of collected data). Prevalence rates were calculated and compared for different areas using Pearson Chi-Square tests. Age-standardized z-scores of body mass index (z-BMI) were calculated according to WHO references [15]. Log transformation of sIgE and total IgE was used to obtain normally distributed data. Student t-tests were used for comparison the differences between the outcomes studied in the two populations. The associations between the risk factors and development of SPT and reported clinical symptoms of allergy were tested by logistic regression. Linear regression was used for analysis of continuous outcomes which provided estimated regression coefficients (β) and their corresponding 95% CI. In multivariate analysis, we included age and sex as *a priori* confounders, as well as other variables that were previously significant in univariate analyses. We retained in the model those for which there was significant heterogeneity across categories, taking P<0.05 as statistically significant. In the case of collinear variables of risk factors, we fitted each in the absence of the other and retained in the model the variable with the strongest effect. The collected data were analyzed using PASW Statistics for Windows, version 18.0 (SPSS Inc, Chicago, IL, USA) software. The risk factors of atopy to HDM and cockroach were different; we have presented the results for each allergen separately.

## Results

### Demographics and allergic disorders in the study population

Data were collected from 1161 and 513 school children in semi-urban and rural areas, respectively. Children were slightly older (mean age 10.6 years) in the semi-urban compared to rural area (9.7 years) (p, <0.001). The percentage of children reported to have history of wheeze was 4.1% (38 out of 927) and 1.1% (5 out of 453) in the semi-urban and rural areas, respectively. Only 16 out of 927 (2.3%) children in the semi-urban area and none in rural area reported doctor-diagnosed asthma. In the semi-urban area, eczema was reported in 3.1% of the children (Table 1), while no case was reported in the rural area.

**Table 1:** Characteristics of population and allergic disorders in semi-urban and rural areas.

	Semi-urban		Rural		p-value
	N	Result	N	Result	
Age years (mean, SD)	1161	10.6 ± 3.3	513	9.7 ± 3.1	<0.001^$^
Sex (N, %)					
Male	583	50.2	272	53.0	0.29^∞^
Female	578	49.8	241	47.0	

Clinical symptoms of allergy (N, n%)					
Asthma					
History of wheeze	927	38 (4.1)	453	5 (1.1)	**0.0026^∞^**
Diagnosed-asthma by doctor	927	16 (2.3)	453	0	
Wheeze in the last 12 months	927	15 (1.1)	453	3 (0.7)	0.14^∞^
Eczema	927	29 (3.1)	453	0	

Skin prick test reactivity (N, n%)					
HDM	975	132 (13.5)	447	38 (8.5)	**0.0066^∞^**
Cockroach	975	232 (23.8)	447	82 (18.3)	**0.021^∞^**

Specific IgE and Total IgE (geometric mean, 95 CI)					
HDM^#^ (kU/L)	592	0.66 (0.58–0.76)	292	1.13 (0.95–1.34)	**<0.001^$^**
*B. germanica* (kU/L)	592	1.33 (1.15–1.54)	292	1.66 (1.38–2.00)	0.074^$^
Total IgE (IU/ml)	540	1896.9 (1696.8–2120.6)	340	4141.1 (3654.7–4692.3)	**<0.001^$^**

SD: standard deviation. The number of positives (n) of the total population examined (N). ^#^IgE to *Dermatophagoides pteronyssinus* (HDM).

The statistically significant results are given in bold. ^$^P-value derived from Student t-test. ^∞^P-value derived from Chi-Square test.

In the semi-urban area the prevalence of SPT was higher than in the rural area for both HDM (13.5% versus 8.5%; p, 0.007; respectively) and cockroach (23.8% vs 18.3%; p, 0.021; respectively). In contrast, the levels of sIgE to HDM and cockroach as well as total IgE were lower in the semi-urban than in the rural area (Table 1).

Rural population were very homogeneous with regards to socio-economic conditions: parental education was low (89%), farming was the major occupation (95%), 92% (473/513) of houses were made up of bamboo, almost all children had helminth infection and a very high proportion of children (78%) had high load of hookworm infection. In contrast, the semi-urban area demonstrated more heterogeneity with 41% (314/760) of parents having high education, 62% (579/938) being farmers, 43% (143/332) of children having a high load of hookworm infection whereas 87% children had helminth infections.

### Risk factors associated with reported clinical symptoms of allergy

No association was found between measured risk factors and history of wheeze, wheeze in the last 12 months or eczema (data not shown).

### Risk factors associated with skin prick test reactivity

In the semi-urban area, the skin reactivity to HDM was positively associated with high paternal education (OR, 1.68; 95% CI, 1.12–2.51) while a high load of *N. americanus* infection was associated with a reduced risk of HDM skin reactivity (OR, 0.47; 95% CI, 0.23–0.95) (Table 2). The odds for positive skin reactivity to cockroach was significantly lower in children who had access to piped water (OR 0.73; 95% CI, 0.54–0.98) but was higher in children whose parents were farmers (OR 1.52; 95% CI, 1.07–2.17) (Table 2).

**Table 2:** Association between skin reactivity to HDM or cockroach and potential risk factors for atopy in the semi-urban area^a^.

	N	HDM		Cockroach	
		n (%)	OR [95% CI]	n (%)	OR [95% CI]
z-BMI (mean, SD)	975	−1.27 ± 1.05^b^	0.95 [0.80–1.13]	−1.27 ± 1.05^b^	0.93 [0.81–1.07]
Paternal education					
Low	442	46 (10.4)	reference	103 (23.3)	reference
High	411	67 (16.3)	1.68 [1.12–2.51]*	89 (21.7)	0.91 [0.66–1.26]

Maternal education					
Low	581	75 (12.9)	reference	151 (26.0)	reference
High	347	54 (15.6)	1.24 [0.85–1.82]	73 (21.0)	0.76 [0.55–1.04]

Parental job					
Non farmer	300	43 (14.3)	reference	55 (18.3)	reference
Farmer	502	65 (12.9)	0.89 [0.59–1.35]	128 (25.5)	1.52 [1.07–2.17]*

House material					
Bamboo / Wood	703	89 (12.7)	reference	175 (24.9)	reference
Stone	272	43 (15.8)	1.30 [0.87–1.92]	57 (21.0)	0.80 [0.57–1.12]

Water source					
Non piped	445	68 (15.3)	reference	120 (27.0)	reference
Piped	530	64 (12.1)	0.76 [0.53–1.10]	112 (21.1)	0.73 [0.54–0.98]*

Toilet					
No	88	10 (11.4)	reference	27 (30.7)	reference
Yes	887	122 (13.8)	1.24 [0.63–2.47]	205 (23.1)	0.68 [0.42–1.10]

Floor material					
Mud	163	17 (10.4)	reference	47 (28.8)	reference
Cement / ceramic	812	115 (14.2)	1.42 [0.83–2.43]	185 (22.8)	0.73 [0.50–1.06]

Fuel					
Wood	827	116 (14.0)	reference	204 (24.7)	reference
Gas / kerosene	148	16 (10.8)	0.74 [0.43–1.29]	28 (18.9)	0.71 [0.46–1.11]

Using sandals					
No	608	79 (13.0)	reference	146 (24.0)	reference
Yes	313	45 (14.4)	1.12 [0.76–1.67]	75 (24.0)	1.00 [0.72–1.37]

*N. americanus* ^1^					
Low load	180	31 (17.2)	reference	49 (27.2)	reference
High load	135	12 (8.9)	0.47 [0.23–0.95]*	25 (18.5)	0.61 [0.35–1.05]

*A. lumbricoides* ^1^					
Low load	200	28 (14.0)	reference	49 (24.5)	reference
High load	115	15 (13.0)	0.92 [0.47–1.81]	25 (21.7)	0.86 [0.49–1.48]

*T. trichiura* ^2^					
Negative	288	32 (11.1)	reference	67 (23.3)	reference
Positive	190	32 (16.8)	1.62 [0.96–2.75]	51 (26.8)	1.21 [0.79–1.84]

a association based on univariate logistic model. ^b^Mean and standard deviation.^ 1^diagnosed by PCR. ^2^diagnosed by microscopy. The number of positives (n) of the total population examined (N). OR: Odds ratio, CI: Confidence intervals. *P<0.05.

None of the measured socio-economic factors, z-BMI or helminth infection status was associated with skin reactivity in the rural area (Table S1).

### Risk factors associated with total and allergen-specific IgE

The risk factors for sIgE were very different from what was seen for SPT. There was no significant association between risk factors measured and the level of sIgE to HDM in the semi-urban area (Table 3). In the rural area, high load of *N. americanus* infection was associated with higher levels of sIgE to HDM (β, 0.25; p, 0.040) (Table S2).

**Table 3:** Association between specific or total IgE and potential risk factors for atopy in the semi-urban area^a^.

	N	IgE to HDM^#^		IgE to *B. germanica*		N	Total IgE	
		geometric mean(95% CI)	β (95% CI)	geometric mean(95% CI)	β (95% CI)		geometric mean(95% CI)	β (95% CI)
z-BMI (mean, SD)	574	−1.35 ± 1.08^b^	−0.02 [−0.08–0.03]	−1.35 ± 1.08^b^	−0.07 [−0.13–0.01]*	509	−.126 ± 1.03^b^	−0.06 [−0.11–0.02]**

Paternal education								
Low	268	0.63 [0.52–0.77]	reference	1.55 [1.26–1.92]	reference	219	2231.6 [1877.8–2652.1]	reference
High	252	0.68 [0.55–0.85]	0.03 [−0.10–0.16]	1.10 [0.87–1.38]	−0.15 [−0.28–0.02]*	241	1644.5 [1382.7–1955.8]	−0.13 [−0.24–0.03]*

Maternal education								
Low	363	0.69 [0.58–0.82]	reference	1.65 [1.38–1.97]	reference	327	2201.7 [1917.7–2527.8]	reference
High	199	0.60 [0.46–0.78]	−0.06 [−0.19–0.07]	0.94 [0.73–1.22]	−0.24 [−0.38–0.11]***	189	1382.2 [1138.7–1677.9]	−0.20 [−0.30–0.10]***

Parental job								
Non farmer	172	0.53 [0.41–0.69]	reference	0.89 [0.68–1.17]	reference	173	1442.4 [1192.3–1744.9]	reference
Farmer	316	0.72 [0.60–0.88]	0.14 [0.00–0.28]	1.66 [1.36–2.02]	0.27 [0.13–0.42]***	262	2171.2 [1839.3–2562.9]	0.18 [0.07–0.29]**

House material								
Bamboo / Wood	445	0.71 [0.61–0.83]	reference	1.52 [1.29–1.78]	reference	396	2018.9 [1780.8–2288.7]	reference
Stone	147	0.54 [0.40–0.72]	−0.12 [−0.26–0.02]	0.89 [0.65–1.22]	−0.23 [−0.38–0.09]**	144	1598.2 [1261.6–2024.6]	−0.10 [−0.21–0.01]

Water source								
Non piped	257	0.68 [0.55–0.84]	reference	1.49 [1.20–1.85]	reference	247	2016.3 [1706.3–2382.5]	reference
Piped	335	0.65 [0.54–0.79]	−0.02 [−0.14–0.11]	1.22 [1.01–1.48]	−0.09 [−0.21–0.04]	293	1801.8 [1550.2–2094.1]	−0.05 [−0.15–0.05]

Toilet								
No	58	0.63 [0.39–1.00]	reference	1.25 [0.74–2.09]	reference	52	2453.7 [1745.3–3449.7]	reference
Yes	534	0.67 [0.58–0.77]	0.03 [−0.18–0.23]	1.34 [1.15–1.56]	0.03 [−0.18–0.24]	488	1845.6 [1640.1–2076.7]	−0.12 [−0.29–0.04]

Floor material								
Mud	108	0.83 [0.60–1.16]	reference	2.05 [1.49–2.82]	reference	82	2343.0 [1753.7–3130.4]	reference
Cement / ceramic	485	0.63 [0.54–0.73]	−0.12 [−0.28–0.04]	1.21 [1.03–1.42]	−0.23 [−0.39–0.07]**	458	1826.5 [1618.5–2061.2]	−0.11 [−0.24–0.03]

Fuel								
Wood	506	0.65 [0.56–0.75]	reference	1.43 [1.23–1.67]	reference	459	1983.8 [1765.1–2229.5]	reference
Gas / kerosene	86	0.73 [0.47–1.12]	0.05 [−0.12–0.22]	0.87 [0.57–1.33]	−0.22 [−0.39–0.04]*	81	1471.8 [1047.8–2067.3]	−0.13 [−0.26–0.01]

Using sandals								
No	376	0.70 [0.59–0.84]	reference	1.46 [1.22–1.76]	reference	302	2027.8 [1751.5–2347.7]	reference
Yes	168	0.64 [0.49–0.82]	−0.04 [−0.18–0.09]	1.16 [0.89–1.51]	−0.10 [−0.24–0.04]	182	1771.6 [1450.3–2164.1]	−0.06 [−0.16–0.05]

*N. americanus* ^1^								
Low load	173	0.62 [0.47–0.83]	reference	1.01 [0.76–1.35]	reference	98	1267.4 [954.3–1683.3]	reference
High load	133	0.68 [0.51–0.89]	0.04 [−0.14–0.21]	1.49 [1.13–1.96]	0.17 [−0.01–0.34]	90	2985.7 [2333.0–3820.9]	0.37 [0.21–0.54]***

*A. lumbricoides* ^1^								
Low load	192	0.57 [0.44–0.74]	reference	1.00 [0.77–1.30]	reference	121	1535.5 [1199.9–1964.9]	reference
High load	114	0.80 [0.59–1.09]	0.15 [−0.03–0.33]	1.63 [1.21–2.18]	0.21 [0.03–0.39]*	67	2833.5 [2065.9–3886.1]	0.27 [0.09–0.44]**

*T. trichiura* ^2^								
Negative	240	0.63 [0.51–0.79]	reference	1.17 [0.93–1.47]	reference	191	1865.2 [1539.0–2260.7]	reference
Positive	158	0.75 [0.56–1.00]	0.07 [−0.09–0.23]	1.47 [1.13–1.92]	0.10 [−0.05–0.26]	96	2043.4 [1590.5–2625.4]	0.04 [−0.10–0.18]

a association based on univariate logistic model. ^b^Mean and standard deviation. The total population examined (N). ^#^IgE to *Dermatophagoides pteronyssinus* (HDM). ^1^diagnosed by PCR. ^2^diagnosed by microscopy. β (beta): estimate regression coefficients. CI: Confidence intervals. *P<0.05, **P<0.01, ***P<0.001.

The examination of cockroach sIgE revealed that in the semi-urban area, high parental education, high socioeconomic status (SES) as represented by housing material, cooking fuel and type of floor as well as high z-BMI showed a significantly negative association with sIgE to cockroach. The high load of *A. lumbricoides* and having farmer parents were associated with high levels of sIgE to cockroach (Table 3). The levels of cockroach sIgE tended to be higher in children with high load of *N. americanus* infection in both semi-urban and rural areas (Table 3 and Table S2).

With respect to the levels of total IgE, in the semi-urban but not in the rural area elevated levels of total IgE were associated with having farmer families as well as high load of *N. americanus* or *A. lumbricoides* infections (Table 3). High maternal education was associated with lower levels of total IgE in both semi-urban (β, −0.20; p, <0.001) and rural (β, −0.15; p, 0.046) areas. The levels of total IgE were significantly lower in children with higher z-BMI (β, −0.06; p, 0.010) in the semi-urban area (Table 3). In rural area, using gas/kerosene as cooking fuel was associated with lower levels of total IgE (β, −0.32; p, 0.030) (Table S2).

### Multivariate analysis

In the semi-urban area, multivariate analysis revealed that *N. americanus* infection tended to reduce the risk of skin reactivity to HDM (adjusted OR, 0.46; 95% CI, 0.21–1.00; p, 0.051) (Table 4). In this model, paternal education did not remain as a significant predictor of skin reactivity to HDM. Cockroach skin reactivity was increased in children with farmer parents (adjusted OR, 1.53; 95% CI, 1.07–2.20; p, 0.019), but the negative association with piped water was no longer significant (Table 4).

**Table 4:** Association between skin reactivity, specific IgE or total IgE and potential risk factors for atopy in the semi-urban area^a^.

	Skin prick test reactivity		IgE to *B. germanica*	Total IgE
	HDM	Cockroach		
	adjusted OR [95% CI]	adjusted OR [95% CI]	adjusted β (95% CI)	adjusted β (95% CI)
General risk factors				
z-BMI			−0.04 (−0.14–0.06)	−0.01 (−0.10–0.07)
Paternal high education (reference: low)	1.82 [0.89–3.74]			
Maternal high education (reference: low)				−0.03 (−0.20–0.14)
Parental farmer (reference:non farmer)		1.53 [1.07–2.20]*	0.24 (0.04–0.44)*	
Piped water (reference: non piped)		0.84 [0.61–1.18]		

Specific parasite infection				
*N. americanus* (reference: low load)	0.46 [0.21–1.00]^¶^			0.33 (0.16–0.50)***
*A. lumbricoides* (reference:low load)			0.15 (−0.05–0.35)	0.22 (0.03–0.40)*

a Multivariate model adjusted with age and sex. β (beta): estimate regression coefficients. CI: Confidence intervals. *P<0.05, **P<0.01, ***P<0.001, ^¶^P<0.1.

Children from farmer parents had higher levels of sIgE to cockroach (adjusted β, 0.24; p, 0.021) but high z-BMI and *A. lumbricoides* load were no longer associated with sIgE (Table 4). Independently, the levels of total IgE were significantly increased with high load of *N. americanus* as well as high load of *A. lumbricoides* infection (adjusted β, 0.33; p,<0.001: adjusted β, 0.22; p, 0.023, respectively) (Table 4).

Multivariate analysis in the rural area showed that high load of *N. americanus* infection was still associated with high levels of HDM sIgE (adjusted β, 0.27; p, 0.031) after adjusting with *a priori* confounders (Table S3). The levels of total IgE in analysis adjusted for age and sex were significantly lower if kerosene/gas was used as cooking fuel compared to wood (Table S3).

## Discussion

The present study showed higher prevalence of positive SPT in semi-urban compared to rural school children on Flores Island in Indonesia. This was accompanied by more reported allergy symptoms. Our finding on rural versus urban differences are in line with other studies showing higher allergies in urban than in rural areas [3], [16]. Populations in rural areas often have helminth infections, traditional life style and lower socioeconomic status. Here we assessed and determined the relative influence of these different factors. Our major finding in the semi-urban area was that although high paternal education was associated with increased risk of skin reactivity to HDM in univariate analysis, in multivariate it was found that only hookworm infection was an independent protective factor for HDM reactivity. This is consistent with a study in Vietnam [10] which found that helminth infections were independent protective factor for HDM sensitization. The results were very different when cockroach skin reactivity was analyzed: factors associated with HDM SPT were not associated with cockroach SPT as has been reported before [10]. In our study only having farmer parents increased the risk of being cockroach SPT positive. We found no association between skin sensitization to these two aeroallergens and any of the potential risk factors measured when we considered the rural area possibly due to low number of SPT positive subjects and homogeneity regarding high helminth load or socio-economic status (SES) factors, such as, low paternal education was almost universal and all children were of farmer families.

Sensitization in terms of serum IgE to HDM and cockroach as well as total IgE showed entirely different patterns. In contrast to SPT, sIgE as well as total IgE were higher in the rural than in the semi-urban area. Helminth infections were associated with higher levels of total IgE in the semi-urban area and tended to increase sIgE to aeroallergens in both rural and semi-urban areas. The finding of enhanced IgE is in line with several previous studies that have demonstrated that total IgE increased with the presence of helminth infections [3], [17], [18].

Epidemiological studies have shown that high parental education, which is one of the indicators of high SES, is associated with atopy [9], [19]. Our study also found that the prevalence of skin reactivity to HDM was influenced by paternal educational level. However, in our study paternal education disappeared as a significant predictive factor in multivariate analysis, while hookworm infection still remained as an independent protective factor on skin reactivity to HDM in the semi-urban area. Helminth infection has been shown in some studies to be inversely related with allergic skin sensitization [6], [10]. As almost all of the population in rural area had intestinal helminth infection, this may explain the absence of an association between helminth infection and HDM reactivity in rural area.

In our semi-urban population we found that the prevalence of skin reactivity to cockroach was lower in children using piped water and higher in children from farmer parents. Piped water is a marker of higher SES, therefore would be expected to be associated with increased SPT. Two possibilities might explain our finding; (i) bacterial contamination of the water which could affect the immune system and thereby atopic sensitization [20], or (ii) piped water is associated with high SES and lower exposure to cockroach. However, in multivariate analysis only having farmer parents remained significantly associated with SPT reactivity to cockroach. The mechanism whereby being a child of farmer parents could increase risk of cockroach SPT positivity is not fully understood but might be related to increased exposure to this allergen.

In addition to having different profile of risk factors for SPT and IgE, another issue of interest is the finding that in the face of high IgE in rural area low SPT is seen. The reason for this discrepancy could not be addressed in this study but it might be due to the fact that helminth infection can potentially induce the production of false-positive serum IgE through cross-reactivity between helminth and aeroallergens as proposed earlier [21]: this is in line with the findings that sensitization to cockroach may not be driven by true cockroach exposure but by cross-reactive carbohydrate determinants (CCDs) as demonstrated in a study from Ghana (Akkerdaas J et al, manuscript in preparation).

The limitation of this study was the cross sectional design in an area with no data on allergen exposure and we could not examine the relation between risk/protective factors with allergic outcomes in the rural area because it was very homogenous with respect to factors such as helminth infection load and socioeconomic status. In addition, we did not measure atopy to *Blomia tropicalis* which is an important mite allergen source in the tropics. Another limitation is that the use of questionnaires to assess information on the presence of allergic disorders such as asthma and risk factors could make the study to information bias. Misclassification of exposure may also have occurred in the present study since we did not evaluate past helminth infection. However, we used a sensitive technique to measure hookworm and *A. lumbricoides* infection load.

In conclusion, the higher prevalence of SPT to HDM in the semi-urban area might be due to high level of education and low helminth burden compared to the rural area. However, we could not explain the lower prevalence of cockroach allergies in the semi-urban area as we found that having a farming family and proxies for lower socioeconomic status, if anything, were associated with increased risk of allergies to cockroach. Further studies are needed to evaluate the possible risk and protective factors in more detail as well as to check possible cross-reactivity between allergens and helminths. So far, our data provide useful information on environmental as well as socioeconomic factors which can be considered by clinicians and researchers working on prevention, diagnosis and treatment of allergic disorders in low-to-middle income countries.

## Supporting Information

Table S1 Association between skin prick test reactivity and potential risk factors for atopy in the rural area^a^.
(DOC)

Table S2 Association between specific or total IgE and potential risk factors for atopy in the rural area^a^.
(DOC)

Table S3 Association between atopy and potential risk factors for atopy in the rural area^a^.
(DOC)
