# Supplementary material for: Risk Factors Associated with the Development of Atopic Sensitization in Indonesia
Source: PLoS One. 2013 Jun 19;8(6):e67064. doi: 10.1371/journal.pone.0067064 (PMC3686782; doi:10.1371/journal.pone.0067064)
Supplement: Table S1 — Association between skin prick test reactivity and potential risk factors for atopy in the rural areaa. (DOC) [file pone.0067064.s001.doc]

**Table S1**. Association between skin prick test reactivity and potential risk factors for atopy in the rural area^a^

|  | **N** | **HDM** | | **Cockroach** | |
| --- | --- | --- | --- | --- | --- |
|  |  | **n (%)** | **OR [95% CI]** | **n (%)** | **OR [95% CI]** |
| z-BMI (mean, SD) | 447 | -1.68 ± 1.14^b^ | 1.11 [0.83-1.49] | -1.68 ± 1.14^b^ | 0.86 [0.70-1.06] |
| Paternal education |  |  |  |  |  |
| Low | 317 | 26 (8.2) | reference | 59 (18.6) | reference |
| High | 81 | 11 (13.6) | 1.76 [0.83-3.73] | 17 (21.0) | 1.16 [0.63-2.13] |
| Maternal education |  |  |  |  |  |
| Low | 364 | 32 (8.8) | reference | 62 (17.0) | reference |
| High | 74 | 6 (8.1) | 0.92 [0.37-2.27] | 18 (24.3) | 1.57 [0.86-2.84] |
| Parental job |  |  |  |  |  |
| Non farmer | 20 | 0 |  | 2 (10.0) | reference |
| Farmer | 367 | 37 (10.1) | - | 72 (19.6) | 2.20 [0.50-9.68] |
| House material |  |  |  |  |  |
| Bamboo / Wood | 410 | 36 (8.8) | reference | 76 (18.5) | reference |
| Stone | 37 | 2 (5.4) | 0.59 [0.14-2.57] | 6 (16.2) | 0.85 [0.34-2.11] |
| Water source |  |  |  |  |  |
| Non piped | 447 | 38 (8.5) |  | 82 (18.3) |  |
| Piped | 0 | 0 | - | 0 | - |
| Toilet |  |  |  |  |  |
| No | 199 | 19 (9.5) | reference | 36 (18.1) | reference |
| Yes | 248 | 19 (7.7) | 0.79 [0.40-1.53] | 46 (18.5) | 1.03 [0.64-1.67] |
| Floor material |  |  |  |  |  |
| Mud | 340 | 28 (8.2) | reference | 60 (17.6) | reference |
| Cement / ceramic | 107 | 10 (9.3) | 1.15 [0.54-2.45] | 22 (20.6) | 1.21 [0.70-2.08] |
| Fuel |  |  |  |  |  |
| Wood | 430 | 36 (8.4) | reference | 81 (18.8) | reference |
| Gas / kerosene | 17 | 2 (11.8) | 1.46 [0.32-6.63] | 1 (5.9) | 0.27 [0.04-2.06] |
| Using sandals |  |  |  |  |  |
| No | 425 | 37 (8.7) | reference | 76 (17.9) | reference |
| Yes | 22 | 1 (4.5) | 0.50 [0.07-3.82] | 6 (27.3) | 1.72 [0.65-4.54] |
| *N. americanus*^1^ |  |  |  |  |  |
| Low load | 41 | 5 (12.2) | reference | 8 (19.5) | reference |
| High load | 149 | 13 (8.7) | 0.69 [0.23-2.06] | 31 (20.8) | 1.08 [0.46-2.58] |
| *A. lumbricoides*^1^ |  |  |  |  |  |
| Low load | 161 | 16 (9.9) | reference | 35 (21.7) | reference |
| High load | 29 | 2 (6.9) | 0.67 [0.15-3.09] | 4 (13.8) | 0.58 [0.19-1.77] |
| *T. trichiura*^2^ |  |  |  |  |  |
| Negative | 170 | 17 (10.0) | reference | 30 (17.6) | reference |
| Positive | 52 | 4 (7.7) | 0.75 [0.24-2.34] | 12 (23.1) | 1.40 [0.66-2.98] |

^a^association based on univariate model. ^b^Mean and standard deviation. ^1^diagnosed by PCR. ^2^diagnosed by microscopy. CI: Confidence interval
