# Supplementary material for: Risk Factors Associated with the Development of Atopic Sensitization in Indonesia
Source: PLoS One. 2013 Jun 19;8(6):e67064. doi: 10.1371/journal.pone.0067064 (PMC3686782; doi:10.1371/journal.pone.0067064)
Supplement: Table S2 — Association between specific or total IgE and potential risk factors for atopy in the rural areaa. (DOC) [file pone.0067064.s002.doc]

**Table S2**. Association between specific or total IgE and potential risk factors for atopy in the rural areaa

|  | **N** | **IgE to HDM^#^** | | **IgE to *B. germanica*** | | **N** | **Total IgE** | |
| --- | --- | --- | --- | --- | --- | --- | --- | --- |
|  |  | **geometric mean** | **β (95% CI)** | **geometric mean** | **β (95% CI)** |  | **geometric mean** | **β (95% CI)** |
|  |  | **(95% CI)** |  | **(95% CI)** |  |  | **(95%) CI** |  |
| z-BMI (mean, SD) | 290 | -1.75 ± 1.15^b^ | -0.02 [-0.08-0.05] | -1.75 ± 1.15^b^ | 0.02 [-0.05-0.09] | 311 | -1.73 ±1.15^b^ | 0.02 [-0.03-0.07] |
| Paternal education |  |  |  |  |  |  |  |  |
| Low | 200 | 1.12 [0.91-1.38] | reference | 1.73 [1.38-2.18] | reference | 238 | 4178.1 [3590.0-4862.4] | reference |
| High | 56 | 1.13 [0.71-1.78] | 0.00 [-0.20-0.20] | 1.39 [0.90-2.16] | -0.10 [-0.31-0.12] | 66 | 4040.8 [2939.0-5555.5] | -0.01 [-0.16-0.13] |
| Maternal education |  |  |  |  |  |  |  |  |
| Low | 236 | 1.09 [0.91-1.31] | reference | 1.60 [1.30-1.97] | reference | 280 | 4400.4 [3831.5-5053.7] | reference |
| High | 50 | 1.27 [0.76-2.13] | 0.07 [-0.13-0.26] | 1.89 [1.20-2.98] | 0.07 [-0.14-0.29] | 55 | 3116.9 [2299.5-4224.8] | -0.15 [-0.30-0.00]* |
| Parental job |  |  |  |  |  |  |  |  |
| Non farmer | 15 | 0.68 [0.30-1.55] | reference | 0.90 [0.44-1.81] | reference | 15 | 3276.5 [2215.8-4845.1] | reference |
| Farmer | 233 | 1.15 [0.95-1.41] | 0.23 [-0.12-0.58] | 1.71 [1.38-2.13] | 0.28 [-0.09-0.66] | 281 | 4263.9 [3688.2-4929.3] | 0.11 [-0.16-0.39] |
| House material |  |  |  |  |  |  |  |  |
| Bamboo / Wood | 265 | 1.13 [0.94-1.36] | reference | 1.70 [1.39-2.06] | ref | 309 | 4088.4 [3596.0-4648.2] | reference |
| Stone | 27 | 1.05 [0.66-1.68] | -0.03 [-0.29-0.22] | 1.35 [0.73-2.52] | -0.10 [-0.38-0.18] | 31 | 4705.3 [2804.6-7893.9] | 0.06 [-0.13-0.25] |
| Water source |  |  |  |  |  |  |  |  |
| Non piped | 292 | 1.13 [0.95-1.34] |  | 1.66 [1.38-2.00] |  | 340 | 4141.1 [3654.7-4692.3] |  |
| Piped | 0 | 0 | - | 0 | - | 0 | 0 | - |
| Toilet |  |  |  |  |  |  |  |  |
| No | 115 | 0.97 [0.74-1.28] | reference | 1.52 [1.13-2.04] | reference | 141 | 4071.4 [3347.4-4951.9] | reference |
| Yes | 177 | 1.24 [0.99-1.54] | 0.11 [-0.05-0.26] | 1.76 [1.38-2.24] | 0.06 [-0.10-0.23] | 199 | 4191.3 [3558.5-4936.6] | 0.01 [-0.10-0.12] |
| Floor material |  |  |  |  |  |  |  |  |
| Mud | 222 | 1.03 [0.84-1.26] | reference | 1.60 [1.28-2.00] | reference | 256 | 3906.7 [3398.4-4490.9] | reference |
| Cement / ceramic | 70 | 1.48 [1.07-2.04] | 0.16 [-0.02-0.33] | 1.87 [1.36-2.58] | 0.07 [-0.12-0.26] | 84 | 4946.1 [3751.9-6520.3] | 0.10 [-0.02-0.23] |
| Fuel |  |  |  |  |  |  |  |  |
| Wood | 278 | 1.13 [0.95-1.35] | reference | 1.70 [1.40-2.06] | reference | 328 | 4251.4 [3743.5-4828.2] | reference |
| Gas / kerosene | 14 | 1.03 [0.55-1.94] | -0.04 [-0.39-0.31] | 1.08 [0.55-2.09] | -0.20 [-0.58-0.18] | 12 | 2019.0 [1099.1-3708.8] | -0.32 [-0.62--0.03]* |
| Using sandals |  |  |  |  |  |  |  |  |
| No | 281 | 1.13 [0.95-1.34] | reference | 1.63 [1.35-1.97] | reference | 298 | 4248.9 [3728.1-4842.4] | reference |
| Yes | 9 | 1.22 [0.23-6.64] | 0.04 [-0.40-0.47] | 3.71 [1.07-12.83] | 0.36 [-0.11-0.82] | 13 | 3981.0 [1694.4-9353.3] | -0.03 [-0.31-0.25] |
| *N. americanus*^1^ |  |  |  |  |  |  |  |  |
| Low load | 36 | 0.62 [0.41-0.94] | reference | 0.97 [0.55-1.74] | reference | 29 | 3243.7 [2147.5-4899.5] | reference |
| High load | 125 | 1.10 [0.84-1.45] | 0.25 [0.01-0.49]* | 1.76 [1.31-2.36] | 0.26 [-0.02-0.53] | 117 | 4476.4 [3626.0-5526.2] | 0.14 [-0.06-0.34] |
| *A. lumbricoides*^1^ |  |  |  |  |  |  |  |  |
| Low load | 139 | 0.99 [0.76-1.28] | reference | 1.59 [1.19-2.12] | reference | 128 | 4347.4 [3537.0-5343.4] | reference |
| High load | 22 | 0.87 [0.52-1.46] | -0.05 [-0.35-0.24] | 1.27 [0.63-2.53] | -0.10 [-0.43-0.24] | 18 | 3279.9 [2199.5-4891.0] | -0.12 [-0.37-0.12] |
| *T. trichiura*^2^ |  |  |  |  |  |  |  |  |
| Negative | 151 | 1.03 [0.82-1.31] | reference | 1.58 [1.22-2.05] | reference | 137 | 3989.2 [3275.0-4859.1] | reference |
| Positive | 42 | 1.25 [0.77-2.03] | 0.08 [-0.14-0.30] | 1.89 [1.08-3.28] | 0.08 [-0.17-0.32] | 45 | 5812.0 [3917.7-8622.1] | 0.16 [-0.01-0.34] |

^a^association based on univariate logistic model. ^b^Mean and standard deviation. The total population examined (N). ^#^IgE to *Dermatophagoides pteronyssinus* (HDM). ^1^diagnosed by PCR. ^2^diagnosed by microscopy. β (beta): estimate regression coefficients. CI: Confidence intervals. *P < 0.05.
