# Supplementary material for: Risk Factors Associated with the Development of Atopic Sensitization in Indonesia
Source: PLoS One. 2013 Jun 19;8(6):e67064. doi: 10.1371/journal.pone.0067064 (PMC3686782; doi:10.1371/journal.pone.0067064)
Supplement: Table S3 — Association between atopy and potential risk factors for atopy in the rural areaa. (DOC) [file pone.0067064.s003.doc]

**Table S3**. Association between atopy and potential risk factors for atopy in the rural area^a^

|  | **IgE to HDM^#^** | **Total IgE** |
| --- | --- | --- |
|  | adjusted β (95% CI) | adjusted β (95% CI) |
| **General risk factors** | | |
| Fuel kerosene/gas (reference: wood) |  | -0.31 (-0.60- -0.30)* |
| **Specific parasite infection** | | |
| *N. americanus* (reference: low load) | 0.27 (0.03-0.51)* |  |

^a^multivariate model adjusted with age and sex.  ^#^IgE to *Dermatophagoides pteronyssinus* (HDM). β (beta): estimate regression coefficients. CI: Confidence intervals. *P < 0.05.
